# Planned Social Media Ban and Depression and Anxiety in Adolescents

**DOI:** 10.1001/jamanetworkopen.2026.31107

**Published:** 2026-08-28

**Authors:** Anna Bilston, Lyndsay Brown, Xu Qi, Aliza Werner-Seidler, Susanne Schweizer

**Affiliations:** 1 University of New South Wales, Sydney, Australia; 2 Black Dog Institute, University of New South Wales, Sydney, Australia

## Abstract

This cohort study assesses underlying assumptions pertaining to adolescent mental health in the implementation of the Social Media Minimum Age restrictions in Australia.

## Introduction

In response to concerns about the association between growth in adolescent social media use and declines in mental health, on December 10, 2025, the Australian Government introduced Social Media Minimum Age restrictions (SMMAR), requiring social media companies to take reasonable steps to prevent individuals under the age of 16 years holding an account. While reviews of reviews have found only small associations between social media use and adolescent mental health, it is recognized that the unique affordances of social media, such as social comparison and exposure to inappropriate content, represent potential harm for young adolescents during a vulnerable developmental period.^1^ However, many adolescents perceive social media as critical to their social lives, driving mixed community support for the SMMAR.^2^

Given this tension, this study aimed to investigate several assumptions underlying the SMMAR: (1) without an account, adolescents will no longer access social media; (2) social media mental health harms are primarily associated with having an account; and (3) that lower age of first use is associated with greater mental health problems. Using baseline data from an ongoing longitudinal study,^3^ these assumptions were assessed based on adolescents’ intended behaviors in the 6 weeks leading up to the introduction of the SMMAR.

## Methods

The survey study was approved by the University of New South Wales Human Research Ethics Committee (iRECS 9197). Immediately before the implementation of the SMMAR (October 25 to December 9, 2025).^4,5^ and after parental consent, a participant screening interview and adolescent assent, eligible adolescents completed a questionnaire battery capturing social media use profiles and intentions to comply (or not) with the SMMAR (eMethods and eAppendix in Supplement 1), as well as symptoms of anxiety and depression, which were assessed with the 7-item Generalized Anxiety Disorder Scale,^6^ and 8-item Patient Health Questionnaire,^4,5^ respectively. To reduce participant burden and enable confidential responses, all surveys were conducted online. Parents and adolescents were asked to complete questionnaires independently.

Separate linear regression models were used to assess the association between symptoms of anxiety and depression, and the following factors: having ever used social media; age of first use; and having a social media account. Binary logistic regression was used to determine whether symptoms of anxiety and depression were associated with the odds of intending to comply with or circumvent the SMMAR. STROBE reporting guidelines for cross-sectional studies were followed. Statistical analysis was completed from December 2025 to March 2026 in R version 4.4.2 (R Project for Statistical Computing). Alpha threshold was set at *P* < .025 to account for 2 outcomes of interest—anxiety and depression.

## Results

In this cohort study, 422 dyads were screened as eligible, and 365 (86%) adolescents (mean [SD], 12.8 [1.4] years; 13% Asian, 13% multiracial, 66% White; 49% female, 49% male, 1.5% nonbinary) completed the questionnaires. From this cohort, 257 participants (70%) had used social media; 1-in-2 had their own social media account, and the mean age of first use was 11.0 years. Having ever used social media and younger age of first use were significantly positively associated with both anxiety and depression symptoms, and having an account was significantly positively associated with anxiety symptoms (Table).

**Table.** Independent Associations Between Social Media Use Variables and Anxiety and Depression

Outcome	β^a^	SE	95% CI^a^	*t*	*df*	*P* value	*R* ^2^
Anxiety (GAD-7)^b^							
Ever used social media^c^	1.00	0.28	0.45 to 1.55	3.57	361	<.001	0.034
Has account^c^	0.70	0.28	0.15 to 1.26	2.48	355	.014	0.017
Age at first use^d^	−1.23	0.28	−1.79 to −0.68	−4.38	354	<.001	0.051
Depression (PHQ-8)							
Ever used social media^c^	0.62	0.27	0.09 to 1.15	2.30	361	.022	0.014
Has account^c^	0.52	0.27	−0.01 to 1.05	1.92	355	.055	0.010
Age at first use^d^	−0.93	0.27	−1.47 to −0.40	−3.44	354	<.001	0.032

Abbreviations: GAD-7, 7-item Generalized Anxiety Disorder Scale; PHQ-8, 8-item Patient Health Questionnaire.

^a^ Linear regression coefficients (β) and 95% CIs are from separate linear regression models. Coefficients indicate the expected change in anxiety or depression score associated with a 1-SD increase in the factor.

^b^ Anxiety indicates total score on the 7-item Generalized Anxiety Disorder Scale (GAD-7)^6^; depression indicates the total score on the 8-item Patient Health Questionnaire (PHQ-8).^5^

^c^ Participants answered yes or no to whether they had ever used social media or had a social media account.

^d^ Participants indicated the age they first used social media from less than or equal to 3 years and then in single age increments to 15 years.

Only 60 social media users (25%) intended to stop using social media completely following the SMMAR. The remaining 185 (75%) intended to continue using social media: 15 (6%) because they were turning age 16 years; 84 (34%) without an account; and 86 (35%) intended to find a way around the restrictions and continue using a social media account. The odds of intending to comply with the SMMAR did not vary significantly with symptoms of anxiety (odds ratio, 1.22; 95% CI, 0.85-1.78; *P* = .28) or depression (OR, 1.08; 95% CI, 0.75-1.56; *P* = .69).

## Discussion

The finding that in the 6 weeks leading up to the Australian SMMAR 75% of Australian 10- to 15-year-olds intended to continue using social media after the restrictions were introduced is concerning as the current findings broadly support the assumptions underlying the SMMAR. Age of first use and ever having used social media (vs never) were associated with greater symptoms of anxiety and depression. Together these findings are consistent with early adolescence as a period of vulnerability for the adverse mental health factors of social media use,^7^ and support the argument for delaying adolescent access to social media. However, with the majority of adolescents intending to circumvent the SMMAR, it is not clear whether this will be the most effective way to raise age of first exposure.

The current findings should be considered within the limitation of this study, which was conducted among relatively well-off, urban participants, limiting generalizability. Furthermore, intended behavior can deviate from actual behavior, and new laws are better accepted once enacted and established as social norms.^8^ Therefore, assessing actual behavior and mental health following the implementation of the SMMAR in diverse populations is essential.

## Notes

Supplement 1. eAppendix. Survey Questions (Qualtrics) eMethods. Recruiting and Screening

Supplement 2. Data Sharing Statement
