# Supplementary material for: Planned Social Media Ban and Depression and Anxiety in Adolescents
Source: JAMA Netw Open. 2026 Aug 28;9(8):e2631107. doi: 10.1001/jamanetworkopen.2026.31107 (PMC13525455; doi:10.1001/jamanetworkopen.2026.31107)
Supplement: Supplementary file 1 [file jamanetwopen-e2631107-s001.pdf]

Supplemental Online Content

Bilston A, Brown L, Qi X, Werner-Siedler A, Schweizer S. Planned Social Media Ban and Depression and Anxiety in Adolescents. *JAMA Netw Open.* 2026;9(8):e2631107.
doi:10.1001/jamanetworkopen.2026.31107

eAppendix. Survey Questions (Qualtrics)
eMethods. Recruiting and Screening

This supplemental material has been provided by the authors to give readers additional information about their work.

© 2026 Bilston AL et al. *JAMA Network Open*.

Some of the following questions refer to social media and messaging. Examples are:

Social media
e.g. Facebook, Instagram, X, TikTok, Snapchat

and

Messaging
e.g. Messages, WhatsApp, Facebook/Kids Messenger, Telegram, Discord, WeChat

For the purpose of this study, ***You Tube, other internet and gaming sites*** are **NOT** considered ***social media or messaging***.

Import from library   Add new question

Online Social Interaction

SM - ever used

Have you **ever used** **social media?** (e.g. Facebook, Instagram, X, TikTok, Snapchat, etc)

- Yes
- No
- Prefer not to say

------------------------------ Page Break ------------------------------

SM - first use

Display this question

If Have you ever used social media? (e.g. Facebook, Instagram, X, TikTok, Snapchat, etc) Yes Is Selected

What **age** were you when you **first used** **social media**?

15

------------------------------ Page Break ------------------------------

© 2026 Bilston AL et al. *JAMA Network Open*.

SM - # used

Display this question

If Have you ever used social media? (e.g. Facebook, Instagram, X, TikTok, Snapchat, etc) Yes Is Selected

How many **social media** applications do you use **once a week or more**?

(e.g. Snapchat only = 1, Snapchat & Instagram = 2, Snapchat & Instagram & Facebook =3, etc)

[0]

--- Page Break ---

TX - ever

Have you **ever used** a **messaging** application (e.g. Messages, WhatsApp, Facebook Messenger, Kids Messenger, Telegram, Discord, WeChat, etc)?

- Yes
- No
- Prefer not to say

--- Page Break ---

TX - first use

Display this question

If Have you ever used a messaging application (e.g. Messages, WhatsApp, Facebook Messenger, Kids Mes... Yes Is Selected

What **age** were you when you **first used** **messaging**?

[15]

--- Page Break ---

© 2026 Bilston AL et al. *JAMA Network Open.*

Own accounts

Do you have your **own account/profile** that you currently use for ...

	Yes	No	Don't Know	Prefer not to say
**Social Media?** (e.g., Facebook, Instagram, X, TikTok, Snapchat, etc)	○	○	○	○
**Messaging?** (e.g., Messages, WhatsApp, Facebook Messenger, Kids Messenger, Telegram, Discord, WeChat)	○	○	○	○
**Gaming?** (e.g., Fortnight, Roblox)	○	○	○	○

Page Break

SM accounts

Display this question

If Do you have your own account/profile that you currently use for ... Social Media?
 (e.g., Facebook, Instagram, X, TikTok, Snapchat, etc) - Yes Is Selected

On **which** of the following **social media** applications do you have your **own account / profile**?

- ☐ Faceb
- ☐ ook
- ☐ Instagr
- ☐ am X
- ☐ TikTok
- ☐ SnapChat
- ☐ Other(s) (please specify:)
- ☐ Prefer not to say

Page Break

SM Use type

Display this question

If Do you have your own account/profile that you currently use for ... Social Media?
 (e.g., Facebook, Instagram, X, TikTok, Snapchat, etc) - Yes Is Selected

When using **social media**, do you **spend more time** ...

Browsing

Sharing, posting or liking Time spent equally Prefer not to say

Aus Ban - aware

Before this question, **did you know that the Government is banning social media accounts for people under 16 years** of age?

Yes

No

Prefer not to say

Page Break

Aus ban - changes

Display this question

If Before this question, did you know that the Government is banning social media accounts for peopl... Yes Is Selected

Has knowing that there will be a ban **changed your social media use already?**

Yes, I deleted my account(s) and chose to stop using social media based on this

Yes, I deleted my account(s) but I still browse social media anonymously

No, I still have a social media account(s)

No, I don't have a social media account(s) but I still browse social media anonymously

No, I don't use social media anyway

Prefer not to say

Page Break

Aus ban - to change

When it starts, **how will the Government ban** on accounts for under-16-year-olds **change your social media usage?**

- I will stop using social media completely
- I will still browse social media without an account
- I will find a way to maintain an account and still use it
- I will be 16 years old by then and will keep using my social media account
- I don't use social media so nothing will change
- Prefer not to say

© 2026 Bilston AL et al. *JAMA Network Open*.

eMethods. Recruiting and Screening

The survey study was approved by the University of New South Wales Human Research Ethics Committee (iRECS 9197). Immediately before the implementation of the SMMAR (October 25 to December 9, 2025).[4,5] After providing consent they and their adolescent completed an online video screening interview to check they met inclusion criteria: living in Australia, English fluency, and adolescent age of 10 to 15 years. To maximize diversity and range of prior social media exposure, adolescents with and without mobile phones were eligible for inclusion.[6]

© 2026 Bilston AL et al. *JAMA Network Open*.
