# Supplementary material for: Planned Social Media Ban and Depression and Anxiety in Adolescents
Source: JAMA Netw Open. 2026 Aug 28;9(8):e2631107. doi: 10.1001/jamanetworkopen.2026.31107 (PMC13525455; doi:10.1001/jamanetworkopen.2026.31107)
Supplement: Supplementary file 2 [file jamanetwopen-e2631107-s002.pdf]

Data Sharing Statement

Bilston. Planned Social Media Ban and Depression and Anxiety in Adolescents. *JAMA Netw Open*. Published August 28, 2026. doi:10.1001/jamanetworkopen.2026.31107

Data
Data available: Yes
Data types: Deidentified participant data
How to access data: https://osf.io/thky7/overview?view_only=dff43632de894115a3bd9db9048b7d18
When available: With publication

Supporting Documents
Document types: Statistical/analytic code, Informed consent form
How to access documents: https://osf.io/thky7/overview?view_only=dff43632de894115a3bd9db9048b7d18
When available: With publication

Additional Information
Who can access the data: Other researchers who consent to adhering to ethical research practices
Types of analyses: Research that adheres to ethical research practices
Mechanisms of data availability: With investigator support
